# Health-Care Provider Screening for Tobacco Smoking and Advice to Quit — 17 Countries, 2008–2011

**Published:** 2013-11-22

**Authors:** Roberta B. Caixeta, Dhirendra N. Sinha, Rula N. Khoury, James Rarick, Heba Fouad, Edouard Tursan d’Espaignet, Soewarta Kosen, Lin Xiao, Judy Kruger, Luhua Zhao, Samira Asma

**Affiliations:** Pan American Health Organization; South-East Asia Regional Office; European Regional Office; Western Pacific Regional Office; Eastern Mediterranean Regional Office; World Health Organization; National Institute for Health Research and Development, Indonesia; China CDC; Office on Smoking and Health, National Center for Chronic Disease Prevention and Health Promotion, CDC

Tobacco use is the leading cause of preventable mortality in the world (1). Article 14 of the World Health Organization (WHO) Framework Convention on Tobacco Control (FCTC) states that countries should promote cessation of tobacco use and adequate treatment for tobacco dependence (2). Health-care providers asking all patients about their tobacco use and advising tobacco users to quit are evidence-based strategies that increase tobacco abstinence (3). This report examines the proportion of tobacco smokers in 17 countries responding to the Global Adult Tobacco Survey (GATS) who saw a health-care provider in the past year and who reported that a health-care provider asked them about smoking and advised them to quit. Respondents were tobacco smokers aged ≥15 years surveyed during 2008–2011 in Bangladesh, Brazil, China, Egypt, India, Indonesia, Malaysia, Mexico, Philippines, Poland, Romania, Russia, Thailand, Turkey, Ukraine, Uruguay, and Vietnam. The proportion of smokers who had visited a health-care provider during the previous 12 months ranged from 21.6% in Egypt to 62.3% in Poland. Among these, the proportion reporting that a health-care provider asked if they smoked ranged from 34.9% in Vietnam to 82.1% in Romania. Among those screened for tobacco use, those who reported their health-care providers advised them to quit ranged from 17.3% in Mexico to 67.3% in Romania. In most countries, persons aged ≥45 years were more likely to report being screened and advised to quit than were persons aged ≤24 years. Health-care providers should identify smokers and provide advice and assistance in quitting at each visit (3) as an adjunct to effective community interventions (e.g., increased price of tobacco products; smoke-free policies, mass media campaigns, and tobacco quitlines).

GATS is an ongoing, nationally representative, in-person household survey of persons aged ≥15 years (4). GATS was conducted in each of the 17 countries during 2008–2011 using a standardized questionnaire, sample design, data collection method, and analysis protocol to enhance data comparability.* Data were weighted to reflect the noninstitutionalized population aged ≥15 years in each country by sex and age groups. Smokers included persons who currently smoked tobacco† and former smokers who were abstinent for <12 months (5). Only smokers were asked, “Have you visited a doctor or other health-care provider in the past 12 months?” If they had been to see a health-care provider, they were then asked two follow-up questions. The first was, “During any visit to a doctor or health-care provider in the past 12 months, were you asked if you smoke tobacco?” Only those who answered “yes” were then asked, “During any visit to a doctor or health-care provider in the past 12 months, were you advised to quit smoking tobacco?”

Survey sampling and analysis followed standard global protocols but were conducted separately for each country. Overall response rates (number of interviews conducted divided by the number of eligible respondents, including those not interviewed) ranged from 65.1% in Poland (2009–2010) to 97.7% in Russia (2009). Survey sample sizes ranged from 4,250 in Malaysia to 69,296 in India (1). Proportions of smokers who were asked about smoking and advised to quit by a health-care provider were calculated by sex, age group, residence (urban versus rural) and education level. Logistic regression for complex sample designs was used to analyze two dependent variables: 1) whether or not the health-care provider asked if the respondent smokes and, 2) whether or not the health-care provider advised the respondent to quit smoking. The demographic characteristics of current smokers (i.e., sex, age group, residence, and education) were used as independent variables in the models. Estimates and 95% confidence intervals were calculated using statistical software. Differences in proportions were considered to be statistically significant if 95% confidence intervals did not overlap.

With the exception of Poland, the prevalence of smokers who reported that they visited a health-care provider in the past year was <60% in all countries surveyed (Table 1). Health-care provider screening for tobacco smoking during any visit in the past 12 months varied and was highest in Romania (82.1%), Uruguay (76.6%), and Egypt (74.1%) and lowest in Vietnam (34.9%), Indonesia (40.5%), and China (40.8%). Report of health-care providers asking and advising to quit varied across countries and was highest in Romania (67.3%), Egypt (67.0%), and Brazil (57.1%) and lowest in Mexico (17.3%), Vietnam (29.7%), and Ukraine (30.8%).

After multivariate adjustment (Table 2), in five of 17 countries, men were more likely to report being asked and advised to quit than women (China, Egypt, India, Indonesia, Thailand), with adjusted odds ratios (AORs) ranging from 1.6 to 8.5. In 14 of the 17 countries, older smokers (aged 45–64 years) were more likely to report being asked and advised to quit than younger smokers (aged ≤24 years), with AORs ranging from 1.8 to 6.7. In India and Mexico, rural smokers who had a health-care visit were less likely to report being screened for tobacco use than urban smokers.

## Editorial Note

The findings in this report indicate that opportunities exist globally for health-care providers to screen for tobacco use and provide smokers with advice to quit. Health-care providers should screen all patients for tobacco use, and for those who use tobacco, provide advice to quit, offer assistance (i.e., counseling and medications), and arrange for follow-up (3). In January 2004, a unified code of practice on tobacco control for health professionals was adopted and signed by the participants at a WHO informal meeting on health professionals and tobacco control in Geneva, Switzerland, to encourage tobacco use prevention and cessation counseling internationally (6).

This international consensus for promoting effective cessation treatment can be used to further promote these practices in the clinical setting. “Offering help to quit tobacco use” is one of the six key focus areas in WHO’s MPOWER package, which is intended to assist countries with the implementation of the WHO FCTC recommendations for tobacco control. Cessation assistance also is a key part of decreasing tobacco use, which is one of CDC’s 10 winnable battles for public health action.§ The WHO MPOWER package also acknowledges the important role health-care systems play in ensuring that health professionals routinely ask all patients about their tobacco use and provide advice to quit (7). Countries might consider implementing community-based tobacco control policies and interventions that both create an environment in which users can successfully stop and increase the likelihood of cessation, including increasing the price of tobacco products and implementing smoke-free policies, mass media campaigns, and tobacco cessation quitlines; these strategies are particularly important because, in some countries, a minority of smokers visited a health-care provider in the last year (7). Low- and middle-income countries might also consider optimizing population coverage and using health services, promoting community-based interventions, and developing partnerships with health-care systems to support cessation and treatment (8).

What is already known on this topic?

Smokers who quit reduce their risk for developing and dying from tobacco-related diseases. Identification of tobacco use and advice to quit by health professionals increases cessation among smokers. Health-care providers should screen all patients for tobacco use, and for those who use tobacco, provide advice to quit, offer assistance, and arrange for follow-up.

What is added by this report?

The proportion of tobacco smokers responding to the Global Adult Tobacco Surveys during 2008–2011 in 17 countries who saw a health-care provider in the past year and who reported that a health-care provider asked them about smoking ranged from 34.9% in Vietnam to 82.1% in Romania; the proportion who said that they were advised to quit ranged from 17.3% in Mexico to 67.3% in Romania. In five of the 17 countries, men were significantly more likely than women to report that a health-care provider asked about smoking and advised them to quit, with adjusted odds ratios ranging from 1.6 to 8.5. In 14 of the 17 countries, older (aged 45–64 years) compared with younger smokers (aged ≤24 years) were significantly more likely to report that a health-care provider asked or advised them to quit, with adjusted odds ratios ranging from 1.8 to 6.7.

What are the implications for public health practice?

Globally, health-care provider screening for tobacco smoking and advice to quit varies widely, and many opportunities to offer effective cessation treatment to tobacco users are being missed.

Disparities across demographic subgroups (sex, age group, and residence) in screening and cessation advice were observed across countries. Barriers to health-care provider counseling at the provider-level typically include time constraints, lack of reimbursement, and lack of professional training (3,9). Data from the 2005 Global Health Professionals Survey indicated that, whereas 87%–99% of health professions students believed they should have a role in counseling patients to quit smoking, only 5%–37% reported that they had received formal training on how to conduct such counseling (10). Reducing barriers to counseling is critical to increasing the number of tobacco users who successfully quit (9). To promote cessation counseling by all health-care providers, their training should include training on smoking cessation counseling (9,10).

The findings in this report are subject to at least six limitations. First, GATS data are self-reported and thus subject to recall bias that might vary across different cultural settings. Second, only screening for tobacco smoking and advice to quit questions were administered; other aspects promoting cessation counseling or medication, reasons for the health-care visit, type of advice provided, or whether follow-up occurred, were not assessed. Third, screening for smoking was only assessed among smokers aged ≥15 years, whereas all adolescents and adults should be screened for tobacco use (3). Fourth, some smokers might have quit before they visited a health-care provider and might, therefore, not have been advised to quit by a health-care provider. Fifth, because response rates ranged from 97.7% to 65.1%, survey respondents might not represent all smokers in some countries. Finally, screening was only assessed among tobacco smokers and not users of other forms of tobacco.

Globally, health-care provider screening for tobacco smoking and advice to quit varies widely, and many opportunities to offer effective cessation treatment to tobacco users are being missed. To reduce the worldwide burden of tobacco use, implementation of WHO FCTC, WHO’s MPOWER package, and further implementation of the cessation guidelines to promote cessation and increase tobacco dependence treatment is warranted.

## Figures and Tables

**TABLE 1:** Percentage of current tobacco smokers* aged ≥15 years who visited a health-care provider during the preceding 12 months and were asked about smoking and advised to quit, by selected characteristics — Global Adult Tobacco Survey, 17 countries, 2008–2011

	Bangladesh (2009†)		Brazil (2008)		China (2010)		Egypt (2009)		India (2009–2010)		Indonesia (2011)	
						
Characteristic	%	(95% CI)	%	(95% CI)	%	(95% CI)	%	(95% CI)	%	(95% CI)	%	(95% CI)
**Percentage of current smokers**	23.0	(21.9–24.2)	17.2	(16.7–17.7)	28.1	(26.7–29.7)	19.4	(18.8–20.1)	14.0	(13.4–14.6)	34.8	(33.2–36.4)
**Percentage of current smokers who visited a health-care provider** §	38.3	(35.0–41.7)	58.8	(57.3–60.3)	30.0	(26.7–33.5)	21.6	(19.9–23.5)	47.3	(45.3–49.4)	30.2	(26.5–34.2)
**Percentage asked by a health-care provider if they smoked** ¶	56.0	(49.9–62.0)	71.0	(69.3–72.6)	40.8	(35.3–46.5)	74.1	(70.7–77.2)	53.0	(50.3–55.7)	40.5	(34.6–46.6)
**Sex**												
Male	55.9	(49.7–61.9)	70.2	(67.7–72.5)	41.7	(36.1–47.6)	75.3	(71.8–78.6)	54.0	(51.2–56.8)	41.6	(35.7–47.8)
Female	64.6	(40.4–83.0)	71.8	(69.5–74.0)	25.5	(18.2–34.5)	35.8	(19.8–55.8)	45.5	(37.6–53.7)	17.9	(9.0–32.4)
**Age group (yrs)**												
15–24	31.3	(20.4–44.9)	54.9	(49.3–60.4)	22.8	(12.2–38.6)	60.9	(46.0–74.0)	31.3	(23.6–40.2)	31.6	(21.6–43.5)
25–44	54.2	(44.1–63.9)	70.2	(67.8–72.5)	34.2	(26.6–42.6)	74.1	(68.8–78.8)	51.3	(47.6–55.0)	38.8	(30.9–47.4)
45–64	69.2	(61.8–75.8)	74.6	(71.9–77.2)	45.9	(39.6–52.4)	76.5	(70.7–81.4)	58.5	(54.1–62.8)	44.2	(37.1–51.4)
≥65	60.1	(45.7–73.0)	81.6	(76.9–85.5)	54.7	(45.9–63.2)	82.7	(71.5–90.1)	63.8	(57.0–70.1)	49.1	(38.3–59.9)
**Residence**												
Urban	52.3	(37.6–66.5)	71.5	(69.7–73.3)	39.4	(33.4–45.8)	74.3	(69.6–78.6)	57.9	(53.8–62.0)	42.1	(34.4–50.2)
Rural	57.4	(51.4–63.2)	67.8	(63.5–71.8)	41.7	(33.8–50.1)	73.9	(69.0–78.2)	51.5	(48.1–54.8)	39.2	(30.9–48.1)
**Education level** **												
Less than primary	56.9	(48.4–65.1)	NA	NA	47.6	(38.9–56.5)	78.2	(73.6–82.2)	54.6	(50.9–58.3)	42.3	(34.2–51.0)
Primary	51.9	(42.6–61.1)	NA	NA	41.0	(31.0–51.7)	72.8	(60.7–82.3)	52.3	(47.4–57.3)	35.4	(25.0–47.3)
Secondary	63.7	(47.3–77.4)	NA	NA	40.0	(33.9–46.5)	69.4	(62.2–75.7)	48.1	(41.9–54.3)	41.3	(33.7–49.2)
University	57.6	(34.6–77.8)	NA	NA	30.0	(20.2–42.1)	67.2	(53.9–78.2)	54.3	(45.0–63.2)	48.6	(36.2–61.2)
**Percentage advised by a health-care provider to quit smoking** ††	52.9	(47.0–58.6)	57.1	(55.3–58.8)	33.9	(29.1–39.0)	67.0	(63.0–70.8)	46.3	(43.6–49.0)	34.6	(29.2–40.5)
**Sex**												
Male	52.7	(46.8–58.5)	55.7	(53.1–58.3)	34.5	(29.6–39.8)	68.4	(64.3–72.3)	47.3	(44.5–50.1)	35.7	(30.3–41.6)
Female	61.6	(38.0–80.7)	58.5	(56.1–60.8)	23.1	(15.0–34.0)	23.4	(12.5–39.4)	38.9	(31.5–46.8)	13.0	(5.6–27.2)
**Age group (yrs)**												
15–24	24.9	(15.5–37.4)	35.1	(30.1–40.4)	17.7	(8.4–33.6)	50.6	(35.7–65.4)	26.1	(19.0–34.8)	27.2	(17.3–39.8)
25–44	50.6	(41.0–60.1)	54.7	(52.1–57.3)	26.6	(21.1–32.9)	66.7	(60.8–72.1)	43.0	(39.4–46.7)	32.3	(25.6–39.7)
45–64	67.3	(59.8–73.9)	64.4	(61.5–67.2)	38.7	(32.4–45.5)	71.3	(64.9–76.9)	52.9	(48.5–57.2)	38.5	(31.5–46.1)
≥65	60.1	(45.7–73.0)	67.3	(61.8–72.4)	48.9	(39.3–58.6)	74.9	(63.6–83.5)	57.9	(51.1–64.4)	43.8	(32.4–55.9)
**Residence**												
Urban	49.0	(35.3–62.9)	57.3	(55.4–59.2)	31.1	(26.2–36.6)	67.6	(62.5–72.2)	50.6	(46.6–54.7)	35.6	(29.2–42.6)
Rural	54.3	(48.4–60.0)	55.8	(51.3–60.2)	35.7	(28.7–43.3)	66.7	(60.9–72.0)	44.9	(41.6–48.3)	33.9	(25.9–42.9)
**Education level**												
Less than primary	53.7	(45.7–61.5)	NA	NA	39.1	(30.1–49.0)	70.8	(64.8–76.0)	47.7	(44.1–51.4)	34.7	(27.3–42.9)
Primary	50.0	(40.8–59.1)	NA	NA	36.9	(27.4–47.6)	70.3	(58.4–80.0)	46.3	(41.4–51.2)	29.8	(20.5–41.2)
Secondary	63.7	(47.3–77.4)	NA	NA	32.4	(27.4–38.0)	62.2	(55.0–69.0)	41.4	(35.7–47.4)	37.5	(29.9–45.7)
University	36.0	(18.4–58.4)	NA	NA	23.2	(14.7–34.6)	58.6	(44.6–71.3)	46.1	(37.1–55.3)	38.3	(26.0–52.4)

	Malaysia (2011)		Mexico (2009)		Philippines (2009)		Poland (2009–2010)		Romania (2011)		Russia (2009)	
						
Characteristic	%	(95% CI)	%	(95% CI)	%	(95% CI)	%	(95% CI)	%	(95% CI)	%	(95% CI)
**Percentage of current smokers**	23.1	(21.2–25.2)	15.9	(14.8–17.1)	28.2	(27.0–29.5)	30.3	(29.0–31.7)	26.7	(25.0–28.4)	39.1	(37.8–40.5)
**Percentage of current smokers who visited a health-care provider**	32.4	(27.9–7.3)	25.0	(22.3–27.8)	24.9	(22.9–27.1)	62.3	(59.6–64.8)	50.4	(46.7–54.0)	54.5	(51.7–57.2)
**Percentage asked by a health-care provider if they smoked**	67.6	(60.0–74.3)	64.7	(59.2–69.9)	67.5	(62.6–72.0)	57.2	(54.2–60.1)	82.1	(77.1–86.3)	45.4	(42.4–48.4)
**Sex**												
Male	67.3	(59.6–74.2)	64.3	(57.5–70.6)	71.6	(66.4–76.3)	58.9	(55.0–62.6)	85.1	(78.5–90.0)	47.7	(44.5–50.9)
Female	75.2	(36.9–94.0)	65.6	(55.5–74.5)	53.4	(43.5–62.9)	55.4	(50.8–59.8)	77.6	(70.9–83.2)	41.3	(35.7–47.1)
**Age group (yrs)**												
15–24	72.5	(47.7–88.4)	56.7	(46.1–66.8)	56.4	(42.5–69.4)	42.5	(33.2–52.4)	66.3	(47.2–81.3)	45.9	(39.9–52.0)
25–44	65.7	(53.8–75.8)	67.6	(59.0–75.2)	71.7	(65.5–77.3)	49.4	(44.8–53.9)	79.4	(73.2–84.4)	39.7	(35.7–43.9)
45–64	68.4	(53.7–80.2)	66.1	(52.5–77.5)	69.7	(61.4–76.8)	65.7	(61.1–70.0)	90.9	(86.0–94.2)	49.2	(44.1–54.3)
≥65	65.5	(43.7–82.3)	78.3	(65.0–87.5)	61.0	(47.6–72.8)	77.4	(67.2–85.2)	87.7	(74.5–94.5)	64.4	(53.9–73.6)
**Residence**												
Urban	65.5	(55.7–74.2)	66.5	(60.1–72.4)	68.0	(60.0–75.0)	58.8	(54.9–62.5)	82.7	(76.9–87.3)	45.8	(42.2–49.4)
Rural	72.8	(64.7–79.7)	54.8	(46.4–63.0)	66.9	(60.9–72.4)	53.8	(49.4–58.1)	81.0	(70.8–88.3)	44.0	(39.6–48.5)
**Education level**												
Less than primary	69.8	(51.3–83.6)	67.4	(56.3–76.8)	61.0	(51.4–69.9)	72.1	(22.4–95.8)	92.6	(77.9–97.8)	0.0	—
Primary	66.4	(52.4–78.0)	66.8	(56.0–76.0)	66.9	(54.5–77.3)	63.4	(55.2–70.9)	89.4	(82.7–93.7)	52.4	(36.9–67.4)
Secondary	69.3	(57.4–79.1)	61.5	(53.8–68.7)	69.8	(62.6–76.1)	55.9	(52.4–59.3)	76.5	(66.9–83.9)	45.7	(42.5–49.0)
University	67.1	(40.1–86.1)	74.8	(59.3–85.8)	73.1	(63.4–80.9)	58.4	(49.7–66.6)	80.8	(73.2–86.6)	44.5	(39.3–49.9)
**Percentage advised by a health-care provider to quit smoking**	52.6	(43.8–61.2)	17.3	(12.3–23.7)	51.6	(47.1–56.1)	41.8	(38.8–44.8)	67.3	(61.9–72.2)	31.8	(29.0–34.7)
**Sex**												
Male	52.2	(43.2–61.0)	17.9	(11.1–27.4)	53.2	(48.0–58.4)	41.2	(37.3–45.2)	68.8	(61.9–74.8)	34.2	(31.1–37.4)
Female	67.4	(31.8–90.1)	16.1	(10.2–24.6)	46.2	(37.1–55.5)	42.5	(37.9–47.2)	65.0	(56.8–72.4)	27.5	(23.1–32.4)
**Age group (yrs)**												
15–24	54.0	(32.3–74.3)	15.7	(8.2–28.2)	43.2	(30.6–56.6)	20.7	(13.9–29.6)	41.7	(25.8–59.5)	24.3	(19.3–30.0)
25–44	47.8	(35.4–60.5)	15.7	(9.4–25.0)	49.3	(42.8–55.9)	32.7	(28.4–37.2)	63.1	(56.2–69.5)	27.0	(23.4–30.9)
45–64	59.3	(45.8–71.6)	20.8	(13.4–30.9)	60.5	(52.4–68.0)	52.0	(47.5–56.5)	80.3	(73.1–85.9)	38.3	(33.1–43.7)
≥65	56.5	(34.6–76.2)	24.5	(13.3–40.6)	48.3	(35.8–61.0)	71.0	(60.8–79.5)	79.8	(66.3–88.8)	59.5	(48.1–69.9)
**Residence**												
Urban	49.6	(38.4–61.0)	17.5	(11.8–25.1)	48.8	(41.9–55.7)	42.2	(38.3–46.3)	66.5	(59.7–72.7)	31.6	(28.3–35.2)
Rural	60.2	(51.9–68.1)	16.4	(10.7–24.2)	54.5	(48.8–60.1)	40.9	(36.9–44.9)	68.8	(59.5–76.7)	32.3	(28.3–36.5)
**Education level**												
Less than primary	65.2	(44.8–81.2)	25.0	(16.2–36.3)	46.2	(37.5–55.1)	63.3	(22.2–91.3)	90.0	(74.8–96.5)	0.0	—
Primary	51.6	(38.9–64.0)	17.9	(9.3–31.5)	56.9	(44.7–68.3)	49.4	(41.4–57.3)	77.5	(68.2–84.6)	45.0	(29.8–61.2)
Secondary	54.0	(42.3–65.2)	17.9	(11.5–26.7)	54.4	(47.5–61.1)	40.4	(37.0–43.9)	59.0	(50.1–67.4)	32.2	(28.9–35.7)
University	43.1	(20.5–68.9)	3.2	(0.6–15.8)	49.7	(39.3–60.2)	41.9	(34.2–50.1)	64.4	(55.4–72.5)	30.4	(25.9–35.3)

	Thailand (2009)		Turkey (2008)		Ukraine (2010)		Uruguay (2009)		Vietnam (2010)	
					
Characteristic	%	(95% CI)	%	(95% CI)	%	(95% CI)	%	(95% CI)	%	(95% CI)
**Percentage of current smokers**	23.7	(22.8–24.7)	31.2	(30.0–32.6)	28.9	(27.7–30.1)	25.0	(23.3–26.6)	23.8	(22.7–24.9)
**Percentage of current smokers who visited a health-care provider**	34.9	(32.7–37.1)	46.9	(44.2–49.7)	32.3	(29.6–35.1)	55.8	(51.8–59.8)	27.2	(25.0–29.5)
**Percentage asked by a health-care provider if they smoked**	60.2	(56.7–63.6)	49.0	(45.8–52.3)	41.7	(36.9–46.6)	76.6	(72.3–80.3)	34.9	(30.9–39.1)
**Sex**										
Male	59.9	(56.1–63.5)	49.1	(45.4–52.9)	43.1	(37.8–48.5)	75.1	(68.2–80.9)	35.3	(31.2–39.7)
Female	63.9	(55.3–71.6)	48.8	(43.3–54.4)	38.2	(29.4–47.8)	77.9	(71.8–83.0)	25.6	(11.8–46.7)
**Age group (yrs)**										
15–24	38.0	(27.0–50.4)	42.0	(33.9–50.5)	36.4	(27.5–46.3)	75.9	(64.1–84.7)	16.8	(8.7–30.0)
25–44	56.5	(50.8–62.1)	45.8	(41.5–50.1)	37.2	(30.9–43.9)	73.7	(67.2–79.3)	32.5	(26.7–38.8)
45–64	66.0	(61.4–70.4)	57.7	(51.8–63.4)	52.2	(43.4–60.9)	83.7	(76.0–89.3)	47.5	(40.2–54.9)
≥65	72.6	(66.6–77.8)	61.0	(47.1–73.3)	62.2	(47.1–75.3)	62.3	(47.7–75.1)	32.6	(22.8–44.3)
**Residence**										
Urban	59.2	(55.2–63.1)	50.6	(46.6–54.5)	39.2	(33.6–45.2)	76.6	(72.1–80.6)	40.9	(35.2–47.0)
Rural	60.7	(56.0–65.1)	44.1	(38.8–49.4)	50.0	(42.5–57.6)	75.8	(65.8–83.6)	31.8	(26.7–37.4)
**Education level**										
Less than primary	69.3	(65.2–73.2)	50.8	(39.6–61.9)	0.0	—	72.2	(56.6–83.8)	36.1	(27.9–45.3)
Primary	57.9	(48.5–66.8)	47.8	(43.1–52.6)	52.9	(33.9–71.0)	76.7	(68.3–83.4)	33.5	(25.7–42.3)
Secondary	49.5	(43.5–55.6)	49.6	(44.2–55.1)	43.0	(37.9–48.2)	77.3	(71.4–82.3)	33.5	(27.3–40.4)
University	55.3	(41.0–68.8)	51.4	(42.8–59.9)	35.7	(26.2–46.5)	75.4	(58.9–86.7)	43.0	(33.7–52.8)
**Percentage advised by a health-care provider to quit smoking**	51.9	(48.4–55.4)	40.7	(37.6–44.0)	30.8	(26.7–35.3)	54.5	(49.4–59.4)	29.7	(25.8–34.0)
**Sex**										
Male	52.3	(48.5–56.0)	42.2	(38.5–46.0)	32.4	(27.8–37.4)	56.7	(49.8–63.3)	30.2	(26.1–34.5)
Female	48.7	(40.1–57.4)	38.0	(32.8–43.5)	26.9	(19.2–36.3)	52.3	(46.0–58.5)	20.3	(8.1–42.4)
**Age group (yrs)**										
15–24	24.2	(15.3–36.0)	33.3	(25.6–42.1)	27.2	(19.7–36.2)	55.6	(43.7–66.9)	14.9	(7.3–28.2)
25–44	48.2	(42.4–54.1)	36.0	(32.1–40.0)	25.0	(19.9–31.0)	48.3	(41.7–54.9)	28.3	(22.7–34.6)
45–64	59.1	(54.5–63.5)	51.5	(45.5–57.4)	41.1	(33.0–49.6)	63.5	(55.2–71.0)	39.6	(32.5–47.1)
≥65	64.8	(58.0–71.0)	60.4	(46.6–72.7)	56.8	(41.1–71.2)	46.0	(31.7–60.9)	27.3	(18.3–38.6)
**Residence**										
Urban	48.8	(44.8–52.8)	42.0	(38.2–46.0)	27.8	(23.0–33.2)	54.5	(49.2–59.7)	33.8	(28.2–39.8)
Rural	53.2	(48.5–57.8)	36.6	(31.6–41.8)	41.1	(33.8–48.9)	53.3	(43.7–62.6)	27.7	(22.8–33.4)
**Education level**										
Less than primary	62.2	(57.8–66.4)	45.0	(33.6–57.0)	0.0	(.–.)	56.5	(43.0–69.1)	28.6	(20.8–38.1)
Primary	50.6	(41.5–59.6)	41.0	(36.4–45.9)	35.9	(19.6–56.3)	58.9	(50.5–66.7)	25.7	(18.8–34.2)
Secondary	39.0	(33.6–44.7)	38.9	(33.9–44.1)	32.4	(27.8–37.4)	52.0	(45.1–58.7)	31.4	(25.3–38.3)
University	46.7	(33.9–60.0)	42.3	(34.2–50.9)	25.0	(17.1–34.9)	46.7	(31.3–62.7)	34.2	(25.5–44.2)

**Abbreviations:** CI = confidence interval; NA = not available.

* Current smokers included former smokers who had abstained for <12 months.

† Year(s) data were collected.

§ During the preceding 12 months.

¶ Among current smokers who had visited a health-care provider during the preceding 12 months.

** Less than primary = no formal education; primary = some primary or completed primary; secondary = some secondary or completed secondary; university = some college/university or more.

†† Among current smokers who had visited a health-care provider during the preceding 12 months, and were asked if they smoked.

**TABLE 2:** Adjusted odds ratios (AORs) for current tobacco smokers* aged >15 years who visited a health-care provider during the preceding 12 months and were asked about smoking and advised to quit — Global Adult Tobacco Survey, 17 countries, 2008–2011

	Bangladesh (2009†)		Brazil (2008)		China (2010)		Egypt (2009)		India (2009–2010)		Indonesia (2011)	
						
Characteristic	AOR	(95% CI)	AOR	(95% CI)	AOR	(95% CI)	AOR	(95% CI)	AOR	(95% CI)	AOR	(95% CI)
**Asked by a health-care provider if they smoked** §												
**Sex**												
Male	0.6	(0.2–1.7)	1.0	(0.8–1.1)	**2.9** ¶	(1.7–4.8)	**6.8**	(3.0–15.2)	**1.7**	(1.2–2.4)	**4.1**	(2.0–8.6)
Female	Ref	Ref	Ref	Ref	Ref	Ref	Ref	Ref	Ref	Ref	Ref	Ref
**Age group (yrs)**												
15–24	Ref	Ref	Ref	Ref	Ref	Ref	Ref	Ref	Ref	Ref	Ref	Ref
25–44	**2.6**	(1.4–5.1)	**1.9**	(1.5–2.5)	1.7	(0.7–3.9)	1.8	(0.9–3.4)	**2.4**	(1.6–3.6)	1.4	(0.8–2.5)
45–64	**5.0**	(2.5–10.0)	**2.4**	(1.9–3.2)	**2.8**	(1.3–6.1)	1.8	(0.9–3.4)	**3.3**	(2.1–5.1)	**1.9**	(1.1–3.3)
≥65	**3.4**	(1.5–7.7)	**3.8**	(2.6–5.4)	**4.5**	(2.0–10.5)	**2.5**	(1.0–6.5)	**4.5**	(2.6–7.5)	**2.6**	(1.3–5.3)
**Residence**												
Urban	Ref	Ref	Ref	Ref	Ref	Ref	Ref	Ref	Ref	Ref	Ref	Ref
Rural	1.2	(0.6–5.4)	0.8	(0.6–1.0)	1.0	(0.6–1.5)	0.9	(0.6–1.3)	**0.7**	(0.6–0.9)	0.9	(0.6–1.5)
**Education level** **												
Less than primary	Ref	Ref	NA	NA	Ref	Ref	Ref	Ref	Ref	Ref	Ref	Ref
Primary	1.1	(0.7–1.8)	NA	NA	0.8	(0.5–1.4)	0.7	(0.4–1.4)	1.0	(0.7–1.2)	0.7	(0.4–1.3)
Secondary	1.5	(0.7–3.2)	NA	NA	1.0	(0.7–1.5)	0.7	(0.4–1.0)	0.8	(0.6–1.1)	1.1	(0.7–1.7)
University	1.1	(0.4–3.5)	NA	NA	0.7	(0.4–1.4)	0.5	(0.3–1.0)	0.9	(0.6–1.4)	1.3	(0.6–2.5)
**Advised by a health-care provider to quit smoking** ††												
**Sex**												
Male	0.6	(0.2–1.7)	0.9	(0.8–1.1)	**2.3**	(1.2–4.3)	**8.5**	(3.9–18.6)	**1.7**	(1.2–2.5)	**4.5**	(1.9–10.9)
Female	Ref	Ref	Ref	Ref	Ref	Ref	Ref	Ref	Ref	Ref	Ref	Ref
**Age group (yrs)**												
15–24	Ref	Ref	Ref	Ref	Ref	Ref	Ref	Ref	Ref	Ref	Ref	Ref
25–44	**3.3**	(1.7–6.4)	**2.2**	(1.7–2.8)	1.6	(0.6–4.0)	1.9	(0.9–3.9)	**2.2**	(1.4–3.4)	1.4	(0.8–2.5)
45–64	**6.7**	(3.3–13.7)	**3.3**	(2.6–4.4)	**2.9**	(1.3–6.6)	**2.3**	(1.1–4.6)	**3.4**	(2.2–5.3)	**2.1**	(1.1–3.8)
≥65	**4.9**	(2.1–11.5)	**3.9**	(2.8–5.3)	**5.0**	(2.0–12.4)	**2.6**	(1.0–6.5)	**4.5**	(2.6–7.7)	**3.1**	(1.4–6.6)
**Residence**												
Urban	Ref	Ref	Ref	Ref	Ref	Ref	Ref	Ref	Ref	Ref	Ref	Ref
Rural	1.20	(0.6–2.3)	0.9	(0.7–1.1)	1.1	(0.6–1.2)	0.9	(0.6–1.2)	**0.8**	(0.6–0.9)	1.0	(0.6–1.6)
**Education level**												
Less than primary	Ref	Ref	NA	NA	Ref	Ref	Ref	Ref	Ref	Ref	Ref	Ref
Primary	1.2	(0.7–1.9)	NA	NA	1.1	(0.6–1.8)	1.0	(0.5–1.8)	1.0	(0.8–1.3)	0.87	(0.5–1.5)
Secondary	1.7	(0.8–3.7)	NA	NA	1.2	(0.7–1.9)	0.8	(0.5–1.2)	0.8	(0.6–1.1)	1.4	(0.8–2.3)
University	0.5	(0.2–1.4)	NA	NA	0.9	(0.4–1.7)	0.6	(0.3–1.1)	0.9	(0.6–1.4)	1.2	(0.6–2.5)

	Malaysia (2011)		Mexico (2009)		Philippines (2009)		Poland (2009–2010)		Romania (2011)		Russia (2009)	
						
Characteristic	AOR	(95% CI)	AOR	(95% CI)	AOR	(95% CI)	AOR	(95% CI)	AOR	(95% CI)	AOR	(95% CI)
**Asked by a health-care provider if they smoked**												
**Sex**												
Male	0.6	(0.1–2.9)	1.0	(0.6–1.6)	**2.2**	(1.4–3.5)	1.2	(0.9–1.5)	1.6	(0.9–2.7)	1.2	(0.9–1.6)
Female	Ref	Ref	Ref	Ref	Ref	Ref	Ref	Ref	Ref	Ref	Ref	Ref
**Age group (yrs)**												
15–24	Ref	Ref	Ref	Ref	Ref	Ref	Ref	Ref	Ref	Ref	Ref	Ref
25–44	0.7	(0.2–2.7)	1.5	(0.8–2.7)	**2.1**	(1.1–3.9)	1.3	(0.8–1.9)	2.0	(0.9–4.3)	0.8	(0.6–1.0)
45–64	0.8	(0.2–2.8)	1.4	(0.6–3.4)	**2.3**	(1.2–4.5)	**2.5**	(1.6–4.0)	**5.0**	(2.1–11.9)	1.1	(0.8–1.6)
≥65	0.5	(0.1–2.6)	**2.7**	(1.0–7.4)	**2.3**	(1.0–5.0)	**4.2**	(2.1–8.5)	2.5	(0.8–7.7)	**2.1**	(1.2–3.4)
**Residence**												
Urban	Ref	Ref	Ref	Ref	Ref	Ref	Ref	Ref	Ref	Ref	Ref	Ref
Rural	1.4	(0.8–2.6)	**0.6**	(0.4–0.9)	1.0	(0.7–1.6)	0.9	(0.7–1.1)	0.7	(0.3–1.5)	0.9	(0.7–1.1)
**Education level**												
Less than primary	Ref	Ref	Ref	Ref	Ref	Ref	Ref	Ref	Ref	Ref	NA	NA
Primary	0.9	(0.3–2.6)	1.0	(0.5–2.2)	1.2	(0.6–2.2)	1.1	(0.1–12.3)	0.7	(0.1–3.4)	NA	NA
Secondary	1.0	(0.3–3.1)	0.9	(0.4–1.9)	1.5	(0.9–2.5)	1.0	(0.1–12.3)	0.3	(0.1–1.1)	NA	NA
University	0.9	(0.2–4.5)	1.5	(0.6–4.0)	1.8	(0.9–3.6)	1.2	(0.1–15.8)	0.3	(0.1–1.1)	NA	NA
**Advised by a health-care provider to quit smoking**												
**Sex**												
Male	0.5	(0.1–2.5)	1.1	(0.5–2.7)	1.4	(0.9–2.1)	1.0	(0.7–1.2)	1.1	(0.7–1.8)	1.2	(0.9–1.5)
Female	Ref	Ref	Ref	Ref	Ref	Ref	Ref	Ref	Ref	Ref	Ref	Ref
**Age group (yrs)**												
15–24	Ref	Ref	Ref	Ref	Ref	Ref	Ref	Ref	Ref	Ref	Ref	Ref
25–44	0.8	(0.3–2.3)	1.1	(0.6–2.1)	1.3	(0.7–2.4)	**1.9**	(1.1–3.1)	**2.5**	(1.2–5.3)	1.2	(0.8–1.6)
45–64	1.2	(0.4–3.5)	1.4	(0.5–3.9)	**2.2**	(1.2–4.2)	**4.2**	(2.5–7.0)	**5.8**	(2.6–12.7)	**1.9**	(1.3–2.8)
≥65	0.7	(0.2–2.9)	1.5	(0.4–4.9)	1.7	(0.8–3.7)	**9.3**	(4.7–18.3)	**4.7**	(1.8–12.3)	**4.5**	(2.5–8.0)
**Residence**												
Urban	Ref	Ref	Ref	Ref	Ref	Ref	Ref	Ref	Ref	Ref	Ref	Ref
Rural	1.5	(0.8–2.6)	0.8	(0.4–1.6)	1.3	(0.9–1.9)	1.1	(0.9–1.4)	0.9	(0.5–1.5)	1.0	(0.8–1.3)
**Education level**												
Less than primary	Ref	Ref	Ref	Ref	Ref	Ref	Ref	Ref	Ref	Ref	NA	NA
Primary	0.6	(0.2–1.8)	0.7	(0.3–1.7)	1.5	(0.8–2.8)	1.1	(0.2–7.2)	0.4	(0.1–1.6)	NA	NA
Secondary	0.7	(0.3–2.1)	0.8	(0.3–1.7)	**1.7**	(1.0–2.7)	1.1	(0.1–7.5)	**0.2**	(0.0–0.7)	NA	NA
University	0.5	(0.1–2.2)	**0.1**	(0.0–0.7)	1.5	(0.8–2.7)	1.3	(0.2–9.6)	**0.2**	(0.0–0.7)	NA	NA

	Thailand (2009)		Turkey (2008)		Ukraine (2010)		Uruguay (2009)		Vietnam (2010)	
					
Characteristic	AOR	(95% CI)	AOR	(95% CI)	AOR	(95% CI)	AOR	(95% CI)	AOR	(95% CI)
**Asked by a health-care provider if they smoked**										
**Sex**										
Male	1.1	(0.7–1.6)	1.0	(0.8–1.3)	1.0	(0.6–1.6)	0.9	(0.5–1.4)	1.9	(0.6–6.2)
Female	Ref	Ref	Ref	Ref	Ref	Ref	Ref	Ref	Ref	Ref
**Age group (yrs)**										
15–24	Ref	Ref	Ref	Ref	Ref	Ref	Ref	Ref	Ref	Ref
25–44	**1.9**	(1.1–3.3)	1.2	(0.8–1.7)	1.1	(0.6–1.7)	0.9	(0.5–1.7)	**2.3**	(1.0–5.0)
45–64	2.4	(0.3–4.4)	**1.9**	(1.2–2.9)	**1.9**	(1.1–3.4)	1.7	(0.8–3.4)	**4.3**	(1.9–9.8)
≥65	**3.0**	(1.5–5.8)	**2.3**	(1.2–4.6)	**2.5**	(1.1–5.3)	0.6	(0.2–1.3)	2.4	(0.9–5.9)
**Residence**										
Urban	Ref	Ref	Ref	Ref	Ref	Ref	Ref	Ref	Ref	Ref
Rural	0.9	(0.7–1.2)	0.8	(0.6–1.0)	1.4	(0.9–2.1)	1.0	(0.5–1.8)	0.7	(0.5–1.0)
**Education level**										
Less than primary	Ref	Ref	Ref	Ref	NA	NA	Ref	Ref	Ref	Ref
Primary	0.8	(0.5–1.3)	1.0	(0.6–1.7)	NA	NA	1.2	(0.5–2.8)	0.9	(0.5–1.6)
Secondary	0.6	(0.4–0.8)	1.1	(0.6–1.9)	NA	NA	1.3	(0.6–2.8)	0.8	(0.5–1.4)
University	0.6	(0.3–1.2)	1.1	(0.6–1.9)	NA	NA	1.2	(0.4–3.3)	1.1	(0.6–2.0)
**Advised by a health-care provider to quit smoking**										
**Sex**										
Male	**1.6**	(1.1–2.3)	1.2	(0.9–1.6)	1.0	(0.6–1.7)	1.2	(0.8–1.6)	1.8	(0.5–6.5)
Female	Ref	Ref	Ref	Ref	Ref	Ref	Ref	Ref	Ref	Ref
**Age group (yrs)**										
15–24	Ref	Ref	Ref	Ref	Ref	Ref	Ref	Ref	Ref	Ref
25–44	**2.6**	(1.4–4.7)	1.1	(0.7–1.7)	0.9	(0.5–1.5)	0.8	(0.5–1.4)	**2.2**	(1.0–5.2)
45–64	**3.3**	(1.7–6.5)	**2.1**	(1.3–3.2)	**1.8**	(1.1–3.1)	1.4	(0.8–2.4)	**3.7**	(1.5–9.0)
≥65	**4.0**	(2.0–8.0)	**3.1**	(1.5–6.0)	**3.2**	(1.4–7.2)	0.7	(0.3–1.5)	2.3	(0.8–6.3)
**Residence**										
Urban	Ref	Ref	Ref	Ref	Ref	Ref	Ref	Ref	Ref	Ref
Rural	1.0	(0.8–1.3)	0.7	(0.5–1.0)	**1.6**	(1.1–2.5)	0.9	(0.6–1.4)	0.8	(0.6–1.2)
**Education level**										
Less than primary	Ref	Ref	Ref	Ref	NA	NA	Ref	Ref	Ref	Ref
Primary	0.8	(0.5–1.3)	0.98	(0.6–1.7)	NA	NA	1.1	(0.6–2.1)	0.8	(0.4–1.6)
Secondary	**0.6**	(0.4–0.8)	0.9	(0.5–1.7)	NA	NA	0.9	(0.5–1.6)	1.1	(0.6–2.0)
University	0.6	(0.3–1.1)	1.0	(0.5–1.7)	NA	NA	0.7	(0.3–1.7)	1.1	(0.6–2.2)

**Abbreviations:** CI = confidence interval; Ref = referent; NA = not available.

* Current smokers included former smokers who had abstained for <12 months.

† Year(s) data were collected.

§ Among current smokers who had visited a health-care provider during the preceding 12 months.

¶ Bolded values indicate statistically significant (p≤0.05) AORs (adjusted for sex, age group, residence, and education level).

** Less than primary = no formal education; primary = some primary or completed primary; secondary = some secondary or completed secondary; university = some college/university or more.

†† Among current smokers who had visited a health-care provider during the preceding 12 months, and were asked if they smoked.

## Footnotes

* Additional information available at http://www.cdc.gov/tobacco/global.

† Respondents who reported currently smoking tobacco on a “daily” or “less than daily” basis.

§ Additional information available at http://www.cdc.gov/winnablebattles.
